# Increased use of high-flow nasal cannulas after the pandemic in bronchiolitis: a more severe disease or a changed physician’s attitude?

**DOI:** 10.1007/s00431-022-04601-w

**Published:** 2022-09-09

**Authors:** Sergio Ghirardo, Giorgio Cozzi, Giovanna Tonin, Francesco Maria Risso, Laura Dotta, Alessandro Zago, Daniela Lupia, Paola Cogo, Nicola Ullmann, Antonella Coretti, Raffaele Badolato, Alessandro Amaddeo, Egidio Barbi, Renato Cutrera

**Affiliations:** 1 Unit of Pediatrics, Department of Medicine, University Hospital S. Maria della Misericordia, Viale San Daniele 27, Udine, Italy; 2 Institute for Maternal and Child Health - IRCCS “Burlo Garofolo”, Trieste, Italy; 3 Department of Medicine, DAME, University of Udine, Udine, Italy; 4 Neonatal Intensive Care Unit, Children’s Hospital, ASST Spedali Civili di Brescia, Brescia, Italy; 5 Department of Pediatrics, Università di Brescia, Istituto di Medicina Molecolare Angelo Nocivelli”, ASST Spedali Civili, Brescia, Italy; 6 University of Trieste, Trieste, Italy; 7 Pediatric Pulmonology & Respiratory Intermediate Care Unit, Clinical, Management and Technology Innovation Research Unit, Academic Department of Pediatrics, Bambino Gesù Children’s Hospital IRCCS, Rome, Italy

**Keywords:** Bronchiolitis, High-flow nasal cannula (HFNC), COVID-19 pandemic, Non-invasive ventilation (NIV), Respiratory syncytial virus (RSV)

## Abstract

After the SARS-CoV-2 pandemic, we noticed a marked increase in high-flow nasal cannula use for bronchiolitis. This study aims to report the percentage of children treated with high-flow nasal cannula (HFNC) in various seasons. The secondary outcomes were admissions for bronchiolitis, virological results, hospital burden, and NICU/PICU need. We conducted a retrospective study in four Italian hospitals, examining the medical records of all infants (< 12 months) hospitalized for bronchiolitis in the last four winter seasons (1 September–31 March 2018–2022). In the 2021–2022 winter season, 66% of admitted children received HFNC versus 23%, 38%, and 35% in the previous 3 years. A total of 876 patients were hospitalized in the study periods. In 2021–2022, 300 infants were hospitalized for bronchiolitis, 22 in 2020–2021, 259 in 2019–2020, and 295 in 2018–2019. The percentage of patients needing intensive care varied from 28.7% to 18%, 22%, and 15% in each of the four considered periods (*p* < 0.05). Seventy-seven percent of children received oxygen in the 2021–2022 winter; vs 50%, 63%, and 55% (*p* < 0.01) in the previous 3 years. NIV/CPAP was used in 23%, 9%, 16%, and 12%, respectively. In 2021–2020, 2% of patients were intubated; 0 in 2020–2021, 3% in 2019–2020, and 1% in 2018–2019.

*Conclusion:* This study shows a marked increase in respiratory support and intensive care admissions this last winter. While these severity indexes were all driven by medical choices, more reliable indexes such as intubation rate and length of stay did not change. Therefore, we suggest that there is a more aggressive treatment attitude rather than a more severe disease.

**Table:** 

**What is Known:** *• COVID-19 pandemic deeply impacted bronchiolitis epidemiology, reducing hospitalizations to onetenth. In the 2021-2022 winter, bronchiolitis resurged to pre-pandemic numbers in Europe.*	
**What is New:** *• Bronchiolitis hospitalization rose much faster in the 2021-2022 winter period, peaking at a higher level. Respiratory supports and high-flow nasal cannula increased significantly compared to the pre-pandemic era.*

**What is Known:**

*• COVID-19 pandemic deeply impacted bronchiolitis epidemiology, reducing hospitalizations to onetenth. In the 2021-2022 winter, bronchiolitis resurged to pre-pandemic numbers in Europe.*

**What is New:**

*• Bronchiolitis hospitalization rose much faster in the 2021-2022 winter period, peaking at a higher level. Respiratory supports and high-flow nasal cannula increased significantly compared to the pre-pandemic era.*

## Introduction

Bronchiolitis represents the first cause of hospitalization in infants and the most common lower respiratory tract viral infection in developed countries. Bronchiolitis is primarily caused by the respiratory syncytial virus (RSV) [1] and usually has a regional-specific seasonality that peaks in the winter season in Italy [2]. The burden of bronchiolitis is relevant for pediatric emergency departments, resulting in overcrowding, economic impact, and an annual spike in the need for respiratory support. In this setting, the use of high-flow nasal cannula (HFNC) has spread worldwide in the past decade [3].

In Italy, the social distancing measures implemented to limit the transmission of the SARS-CoV-2 virus reduced the incidence of bronchiolitis to a tenth in the 2020–2021 winter seasons. These measures had an even more significant impact on RSV epidemiology [4]. In the 2021–2022 winters, many of these measures were removed. In the 2021–2022 winters, we noticed a substantial increase in HFNC in some Italian pediatric wards, in our clinical practice.

The study aimed to describe the percentage of children treated with HFNC, the epidemiology of bronchiolitis, its severity, and hospital burden in the last winter compared to the pandemic year and the previous years in four Italian hospitals.

## Methods

This retrospective multicenter study was performed in four hospitals (three in the north and one in the center of Italy): the Children’s Hospital Institute for Maternal and Child Health IRCCS Burlo Garofolo of Trieste, the Bambino Gesù Children’s Hospital IRCCS, Rome, the ASST Spedali Civili Brescia, and the S. Maria della Misericordia University Hospital, Udine.

As this study used pre-existing, deidentified data, the Institutional Review Board considered this study exempt. According to the Italian law, the Authorization to Process Personal Data for Scientific Research Purposes (Authorization No. 9/2014) declared that retrospective archive studies that use ID codes, preventing the data from being traced back directly to the data subject, do not need ethics approval [5].

We reviewed the electronic registry and medical records of all patients under 1 year of age discharged with a final diagnosis of bronchiolitis. We categorized the disease with ICD9 or ICD10 codes indicating “acute RSV bronchiolitis” or “acute bronchiolitis from other specified organisms” [6] during the last four-season onset periods (SOPs) from September 1 to March 31, respectively. Patients whose diagnosis was not confirmed during hospitalization were excluded. For each patient, we anonymized and then recorded the admission site and date, birth and of discharge date, neonatal weight and gestational age, days spent on oxygen, HFNC, NIV/CPAP, mechanical ventilation, in NICU/PICU, the virus or viruses isolated from the nasopharyngeal swab, and the presence and type of comorbidities. The primary outcome was the percentage of children treated with HFNC during hospitalization, pooling data of ED, clinics, and NICU/PICU. The secondary outcomes were admissions for bronchiolitis, virological results, hospital burden, NICU/PICU need, and other respiratory support. In the four centers, there were no shared indications for HFNC start, procedures of use, and NICU/PICU admission outside clinician choice. All the ED centers involved in the study routinely employ HFNC outside NICU/PICU setting. In contrast, CPAP and NIV are not usually initiated in any of the ordinary wards involved in the study.

### Statistical analysis

We reported categorical variables as numbers and percentages and continuous variables as mean and standard deviation or median and first (Q1) and third quartile (Q3) if not normally distributed. We performed the Fisher exact test for discrete variables to compare the last season with the COVID season and the Kruskal–Wallis test to compare the last season to the two pre-pandemic seasons. We used a Log-rank test to assess the time distribution of hospitalizations, confronting the current season with the previous ones. For categorical variables, we evaluated normality visually, employing the Shapiro–Wilk test. We compared the last season with the pandemic season using the Student test when normally distributed and the Wilcoxon-signed-rank test when not normally distributed for categorical variables. At the same time, we used the ANOVA test to compare the last season with the two pre-pandemic seasons. Using the Log-rank test, we compared the in-hospital and NICU/PICU lengths of stay and various respiratory support. Throughout the study, we considered a confidence interval of 95% statistically significant with a *p* value of 0.05.

## Results

A total of 876 patients were admitted to the four centers in the four SOPs considered. During the last SOP, 300 infants were hospitalized with bronchiolitis; 22 in the COVID SOP, 259 and 295 in each pre-pandemic SOPs. There were no statistically significant differences in patients’ characteristics between years except for the COVID season in which hospitalized patients were, on average, older and for viral epidemiology (see Table 1).

**Table 1:** Patient characteristics; we report in this table the four SOPs (1 September to 31 March)

	2018–2019	2019–2020	2020–2021	2021–2022	*p* value
Patients per year	295	259	22	300	
Rome	130	131	6	142	
Brescia	94	73	9	99	
Trieste	41	19	3	33	
Udine	30	36	4	26	
Age at admission (days)	47.5 (32–113)	59 (33–121)	115 (39–288.5)	48 (25–100)	0.002*
Gestational age (weeks)	38 ± 2.4	38 ± 2.5	37 ± 4.8	38 ± 2.8	0.8
Birth weight (kg)	3.2 ± 0.6	3.1 ± 0.6	2.8 ± 1.0	3.2 ± 0.7	0.6
RSV cases	179 (61%)	175 (68%)	1 (4%)	240 (80%)	< 0.001*
Rhinovirus cases	32 (11%)	37 (15%)	8 (57%)	56 (19%)	< 0.001**

Data are expressed as median reporting in brackets, the first and third quartile or median and standard deviation using the ± symbol if data are normally distributed

^*^The difference is statistically significant between the 2020–2021 season and the others

**The difference is statistically significant between the 2020–2021 season and the others

In the last SOP, 197 patients (66%) received HFNC treatment, 5 patients (23%) the HFNC in the COVID SOP (*p* < 0.001), and 99 (38%) and 102 (35%) the HFNC treatment in the two pre-pandemic SOPs (*p* < 0.001).

**Fig. 1:**
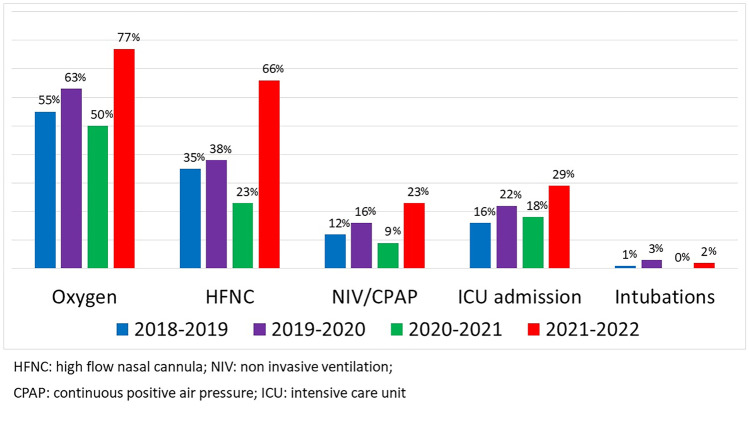
Respiratory support and NICU/PICU needs expressed as percentages in the fourth season onset periods

42 (16%)In the most recent SOP, HFNC, if used, were employed for a median of 4 (Q1–Q3 2.6) days, 1.5 (Q1–Q3 1–3.5) days in COVID SOP (*p* < 0.001), and 4 (Q1–Q3 2–6) and 3 (Q1–Q3 2–5) days respectively in each of the two pre-pandemic SOPs (*p* < 0.001). Data about respiratory support and NICU/PICU admission are reported in Table 2 and graphically in Fig. 1. In the last SOP, HFNC were less used in the two centers with the most cases (*p* = 0.015), as shown in Table 2.

**Table 2:** Oxygen need and respiratory supports for patients hospitalized for bronchiolitis

	2018–2019	2019–2020	2020–2021	2021–2022	*p* value
Patients needed oxygen therapy	161 (55%)	164 (63%)	11 (50%)	231 (77%)	< 0.01*
Length of treatment (days)	4 (2–6)	5 (3–7)	2.5 (1.75–4)	4 (3–6)	*p* = 0.015
Respiratory support: *n* (%)	110 (37%)	106 (41%)	6 (27%)	207 (69%)	< 0.001**
HFNC: *n* (%)	102 (35%)	99 (38%)	5 (23%)	197 (66%)	*p < 0.001*
CPAP/NIV: *n* (%)	36 (12%)	42 (16%)	2 (9%)	68 (23%)	*p = 0.003*
Mechanical ventilation: *n* (%)	3 (1%)	8 (3%)	0	6 (2%)	*p = 0.26*
Respiratory support by center: *n* (%)	110 (37%)	106(41%)	6 (27%)	207 (69%)	*p* < 0.001**
HFNC: *n* (%)	102 (35%)	99 (38%)	5 (23%)	197 (66%)	*p* < 0.001
Rome	-37 (28%)	-42 (32%)	-1 (17%)	-91 (64%)	*p* = 0.01***
Brescia	-42 (45%)	-33 (45%)	-3 (33%)	-57 (57%)	
Trieste	-6 (15%)	-5 (26%)	- 1 (33%)	-28 (84%)	
Udine	-17 (57%)	-19 (52%)	-0	-21 (81%)	
CPAP/NIV: *n* (%)	36 (12%)	42 (16%)	2 (9%)	68 (23%)	*p* = 0.003
Rome	-12 (9%)	-22 (17%)	-0	-23 (16%)	*p* = 0.04***
Brescia	-18 (19%)	-11 (15%)	-2 (22%)	-31 (31)	
Trieste	-3 (7%)	-3 (16%)	-0	-9 (27%)	
Udine	-3 (10%)	- 6 (17%)	-0	-5 (19%)	
Respiratory supports duration: days					
HFNC	3 (2–5)	4 (2–6)	1.5 (1–3.5)	4 (2–6)	*p* = 0.6
CPAP/NIV	3 (2–5)	3 (2–5)	1 (1–4)	4 (2–6)	*p* = 0.8
Mechanical ventilation	5; 1; 8	4.5 (2.5–6.5)	0	3 (2–8)	*p* = NA
Patients admitted to NICU/PICU, *n* (%)	46 (16%)	57 (22%)	4 (18%)	86 (29%)	*p* = 0.013
NICU/PICU length of stay days	4 (3–7)	5 (3.5–8)	2.5 (1–5)	6 (4–7)	*p = 0.3*
In-hospital length of stay (days)	4 (3–7)	5 (3–8)	4 (3–7)	6 (4–8)	*p* = 0.09***

Data are expressed as median reporting in brackets, the first and third quartile or median and standard deviation using the ± symbol if data are normally distributed

*NICU* neonatal intensive care unit, *PICU* pediatric intensive care unit, *HFNC* high-flow nasal cannula, *CPAP* continuous positive air pressure, *NIV* non-invasive ventilation

^*^The difference is statistically significant between the 2021–2022 season and the others

^**^The difference is statistically significant between the 2021–2022 season and the others

***The difference is statistically significant between the 2019–2020 season and the others

**Fig. 2:**
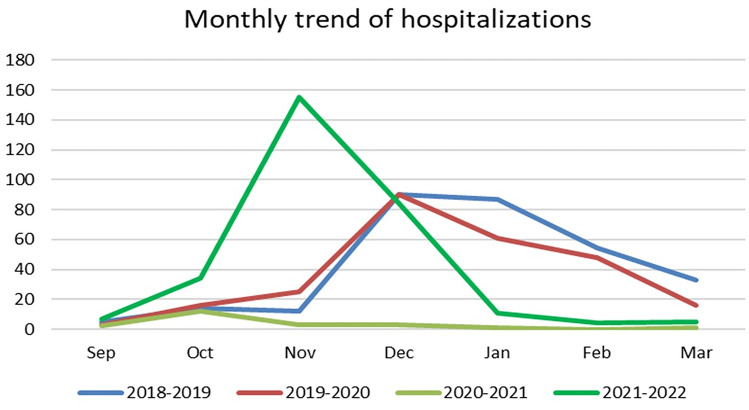
Patients hospitalized each month during the fourth season onset periods

Applying the Log-rank test, we noticed statistically significant anticipation of the bronchiolitis onset in the last SOP: 83 (Q1–Q3 71–97) days from the beginning of the SOP (September 1) compared to the COVID SOP (49.5 Q1–Q3 35.5–84 days; *p* = 0.04) and both pre-pandemic SOPs, with 121 (Q1–Q3 103–152) days and 130 (Q1–Q3 110–161) days (*p* < 0.001), respectively. In the last SOP, 7% (27 patients) of the total were hospitalized after January 1, compared to 9% (2 patients) after this date in the COVID SOP (*p* = 1.00); in the pre-pandemic SOPs, 48% (124 patients) and 59% (174 patients) were hospitalized after January 1, respectively (*p* 0.001) (see Fig. 2 for the monthly distribution of hospitalizations).

## Discussion

This study shows a remarkable increase in the use of HFNC, in terms of absolute numbers and percentage of patients, by Italian pediatricians in the last winter. In the last SOP, 66% of children admitted for bronchiolitis received HFNC versus 23% in the pandemic SOP and 38% and 35% in the two pre-pandemic selected SOPs.

This year, hospitalizations for bronchiolitis increased sharply (13 times vs the 2020–2021 season), reaching the pre-pandemic incidence as a probable effect of social distancing measures withdrawal [4].

In this last SOP, 80% of cases of bronchiolitis were sustained by RSV, presenting a statistically significant increase compared to the pandemic and pre-pandemic periods [7, 8]. While RSV has been reported to lead to more prolonged hospitalizations and possibly to a more severe disease course [9, 10], we believe this percentage can explain only a minor part of the striking increase in the HFNC surge observed in the last SOP. In addition, hospitalizations for bronchiolitis increased in a shorter time in the latter SOP, peaking earlier in November with a high almost double compared to the pre-pandemic period. This distribution of hospitalization resulted in the worst burden on paediatric EDs and more pronounced hospital crowding than usual [11].

Over the past season, the bronchiolitis burden grew in terms of the need for oxygen and respiratory support, with a statistically significant increase in the number of infants who needed it compared to the pre-pandemic SOPs. The most remarkable rise concerned HFNC use, from 35–38% of the pre-pandemic period to 66% of the latter SOP. Also, NIV/CPAP use nearly doubled compared to the same periods, while the NICU/PICU was slightly less remarkable. These three aspects — increased oxygen, other respiratory support, and admission to NICU/PICU — could suggest a more severe course of the disease, which could be related to the higher prevalence of RSV in the last SOP [9]. However, studies linking RSV and bronchiolitis severity are controversial [12] as all of these aspects are clinically driven by physicians’ overall impression [13]. Remarkably, the most reliable and less clinician impression–driven aspects did not differ between the latter SOP and the pre-pandemic SOPs, such as the need for invasive mechanical ventilation, length of hospital stay, and NICU/PICU admissions [14]. Death did not occur in this study. Furthermore, we found that the increase in HFNC, NIV/CPAP use, and NICU/PICU admission could be traced to a worldwide trend in augmenting the intensity of bronchiolitis care over the past 14 years. However, the stagnation in the hospitalization rate and population characteristics [15] suggests a more aggressive attitude of clinicians rather than a more severe disease [14]. According to the first studies, HFNC initially appeared to prevent intubation, especially if started early [16, 17]. While these results led to widespread use of HFNC in the pediatric ED setting, they were eventually not confirmed and, even denied [18]. It is speculated that the global spread of the HFNC in paediatric ED settings may contribute to treatment escalation since the disease is perceived as more severe if patients do not respond to this treatment. Therefore, in this perspective, HFNC could even become a risk factor for invasive mechanical ventilation escalation [14].

Overall, we suggest that the increased use of respiratory support and NICU/PICU admission observed in our study was due to several factors. Less confident pediatricians with the clinical presentation of bronchiolitis, nearly absent during COVID SOP, and a global trend towards more aggressive supportive care caused an increase and even abuse of HFNC treatment. The recent history of medical treatments and monitoring of bronchiolitis was characterized by a constant “*less is more*” trend. No routine investigations (X-rays, swabs) were routinely needed, and all treatments proposed through the decades for the disease were substantially ineffective [19]. Continuous SpO_2_ monitoring in children with mild conditions who were not receiving oxygen was discouraged because its use was not related to an evident increase in the quality of care, leading to unjustified prolonged length of stay [13]. In this mainstream, a 68% use of HFNC, as revealed in this study, appears inappropriate, suggesting that Italian pediatricians should consider more stringent criteria for its usage.

Our investigation had some limits, it was performed only in Italian hospitals, and due to its retrospective nature, we could not rule out possible misclassifications of patients in clinical records, the absence of standardized clinical scores in the clinical records, and decision-making of the patients. Moreover, we have no data about the setting (standard departments or NICU/PICU) in which respiratory support was started.

In conclusion, we demonstrated a striking increased use of HFNC by Italian pediatricians in the setting of a marked recovery in hospitalizations for bronchiolitis in the last winter season with a more pronounced prevalence of RSV infections than before the COVID-19 pandemic period. This escalation to more aggressive support treatment should be considered a sign of non-evidence-based overtreatment, and pediatricians should further question their attitudes concerning this disease treatment.

## Data Availability

Complete data of the article are at disposal after proper request to the corresponding author.

## Code availability

We used excel and R for data analysis.
